# Kinase-dependent regulation of ciliary protein transport and its implications for therapy

**DOI:** 10.3389/fmolb.2025.1638737

**Published:** 2025-06-25

**Authors:** Taro Chaya, Yuri Ayano, Takahisa Furukawa

**Affiliations:** Laboratory for Molecular and Developmental Biology, Institute for Protein Research, The University of Osaka, Osaka, Japan

**Keywords:** CILK1, ICK, MAK, ciliary tip, retinitis pigmentosa

## Abstract

Primary cilia are evolutionarily conserved microtubule-based structures that extend from the surfaces of many different cell types and decode a wide range of extracellular chemical and physical stimuli. Ciliary defects cause human diseases, termed ciliopathies, which are characterized by a variety of symptoms, such as developmental and sensory abnormalities. The formation and function of primary cilia depend on intraflagellar transport (IFT), which is a bidirectional protein transport system coordinated by three multi-subunit protein complexes with kinesin and dynein motors along the ciliary axoneme. Accumulating evidence has demonstrated that several serine-threonine kinases play key roles in the regulation of IFT. Here, we review the current understanding of the roles of these kinases during the IFT process, as well as their regulatory mechanisms, physiological and pathophysiological significance, and potential to treat ciliopathies and age-related obesity.

## Introduction

Primary cilia are hair-like organelles that protrude from nearly all cell types and perform diverse sensory functions. Cilia and flagella are evolutionarily conserved membranous structures that have a wide range of functions, including motility and sensation, among species from unicellular organisms to humans. Primary cilia consist of a microtubule-based axoneme core that extends from a modified centriole, the basal body (Gerdes et al., 2009; Malicki and Johnson, 2017). The ciliary membrane and cilioplasm are separated from the plasma membrane and cytoplasm, respectively, by the transition zone and transition fibers (Garcia-Gonzalo and Reiter, 2017). A variety of receptors, ion channels, and their downstream signaling molecules localized to the primary cilia detect and decode extracellular stimuli including light, odorants, and Hedgehog morphogens (Mill et al., 2023). For example, retinal photoreceptor cells develop outer segments, which are specialized primary cilia that contain phototransduction components to receive light and convert it into electrical signals (Wang and Deretic, 2014). Therefore, primary cilia are recognized as hubs for multiple signal transduction pathways. Ciliary dysfunction causes human diseases called ciliopathies, which are characterized by a wide range of pathologies including polydactyly, craniofacial abnormalities, brain malformation, intellectual disability, obesity, diabetes, polycystic kidney disease, anosmia, hearing loss, and retinal degeneration (Fliegauf et al., 2007; Nigg and Raff, 2009; Anvarian et al., 2019).

## Intraflagellar transport

**FIGURE 1:**
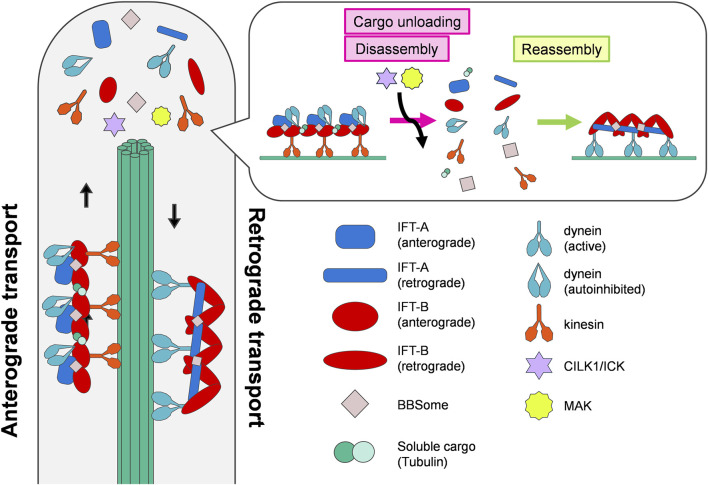
IFT turnaround at the tip of cilia. IFT is a microtubule-based bidirectional cargo transport in cilia coordinated by anterograde and retrograde trains. The anterograde trains unload their cargoes, disassemble, and reassemble into morphologically distinct retrograde trains at the ciliary tips. The ciliary kinases CILK1 and MAK promote cargo unloading and disassembly of anterograde trains.

The formation, maintenance, and function of cilia rely on intraflagellar transport (IFT), bidirectional protein trafficking coordinated by three protein complexes, IFT-A, IFT-B, and BBSome, with molecular motors along the ciliary axoneme (Figure 1). They form highly repetitive polymers called IFT trains, which import and export ciliary proteins, and deliver ciliary cargoes along the axoneme in both anterograde and retrograde directions (Rosenbaum and Witman, 2002; Lechtreck, 2015; Nachury, 2018; Nakayama and Katoh, 2018; Pigino, 2021). The kinesin-2 motor drives anterograde transport from the base to the tip of the cilium, whereas the cytoplasmic dynein-2 motor drives retrograde transport from the tip to the base (Rosenbaum and Witman, 2002; Nachury, 2018). At the tip of the cilia, IFT trains unload their cargoes and subsequently disassemble and reassemble for turnaround and retrograde transport (Chien et al., 2017). Mutations in the genes encoding components of IFT trains have been reported to cause human ciliopathies, including Bardet-Biedl syndrome (BBS) and Joubert syndrome (Reiter and Leroux, 2017).

A recent visualization of retrograde trains in *Chlamydomonas* by cryo-electron tomography provided structural insights into the transition from anterograde to retrograde transport (Figure 1) (Lacey et al., 2024). IFT-A and IFT-B complexes adopt different conformations in anterograde and retrograde transport. At the ciliary tips, anterograde trains unload their cargoes and remodel into retrograde trains. During this process, the anterograde train depolymerizes and the IFT-A and IFT-B complexes reassemble into morphologically distinct retrograde trains (Pedersen et al., 2006; Pigino et al., 2009; Chien et al., 2017; Lacey et al., 2024). Autoinhibited dynein-2 motors are released from the anterograde train and transformed into an open conformation (Jordan et al., 2018). The remodeled IFT complexes bind to activated dynein-2 motors and cargoes to conduct retrograde transport.

## Regulation of intraflagellar transport by serine-threonine kinases

Several serine-threonine kinases are known to play key roles in the regulation of IFT. Before anterograde transport, IFT-A and IFT-B components are recruited to the basal body to assemble into anterograde trains. Deficiency of Tau tubulin kinase 2 (Ttbk2), a serine-threonine kinase localized to basal bodies, in mouse embryonic fibroblasts (MEFs) decreases the accumulation of IFT-A and IFT-B components at the basal body, resulting in shortening or absence of cilia (Goetz et al., 2012; Nguyen and Goetz, 2023). In contrast, depletion of the casein kinase 2 (CK2) catalytic subunit (Csnk2a1), a negative regulator of Ttbk2, in MEFs increases the basal body localization of IFT-A and IFT-B components and ciliary length (Loukil et al., 2021), suggesting that the two serine-threonine kinases TTBK2 and CK2 modulate the initial phase of IFT, although the underlying mechanisms remain unclear.

Another two serine-threonine kinases intestinal cell kinase (ICK), also known as ciliogenesis-associated kinase 1 (CILK1), and male germ cell-associated kinase (MAK) have been shown to be critical regulators of IFT turnaround step at the ciliary tip (Hesketh et al., 2022; Nachury, 2022; Lacey and Pigino, 2025) (Figure 1). CILK1 and MAK are evolutionarily conserved mitogen-activating protein kinase-like kinases that show high homology, especially in their catalytic domains (Miyata and Nishida, 1999; Togawa et al., 2000; Shinkai et al., 2002). *Cilk1* is ubiquitously expressed in multiple tissues, whereas *Mak* is preferentially expressed in the retina and testis (Tsutsumi et al., 2018). In contrast to their distinct expression patterns, these kinases show a similar subcellular localization. CILK1 and MAK localize mainly to the ciliary tip in cultured cells and to the distal region of ciliary axonemes in retinal photoreceptor cells (Omori et al., 2010; Chaya et al., 2014; Chaya et al., 2024). Loss of CILK1 function causes dysregulation of ciliary length, impaired Hedgehog signaling, and accumulation of IFT-A, IFT-B, and BBSome components at the ciliary tips (Broekhuis et al., 2014; Chaya et al., 2014; Moon et al., 2014; Okamoto et al., 2017; Nakamura et al., 2020). Since ciliary length is controlled by IFT, regulation of IFT has been proposed to be linked to ciliary length regulation (Ishikawa and Marshall, 2011). *Mak*-deficient mice exhibit elongated photoreceptor ciliary axonemes with accumulation of IFT-A and IFT-B components at the distal portion (Omori et al., 2010; Chaya et al., 2024). These observations propose a model in which CILK1 and MAK promote the disassembly of anterograde trains in the turnaround process. This model is supported by a recent study showing that *Caenorhabditis elegans* (*C*. *elegans*) DYF-5, an ortholog of CILK1 and MAK, plays a key role in regulating the turnarounds of IFT trains at the ciliary tip, using fluorescence imaging and single molecule tracking (Mul et al., 2025).

**FIGURE 2:**
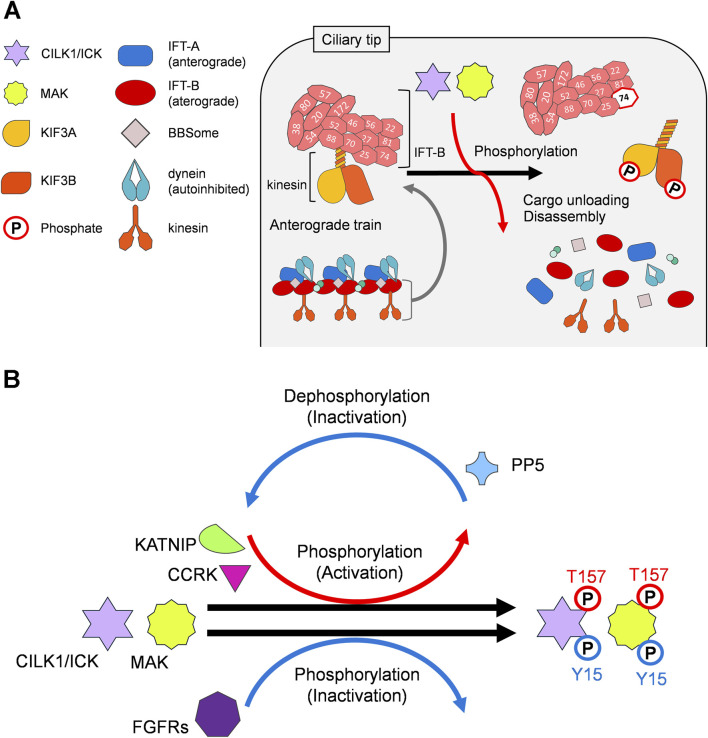
Ciliary kinases CILK1 and MAK in the regulation of IFT. **(A)** Working model of the regulation of cargo unloading and IFT train disassembly at the tip of cilia by CILK1 and MAK through phosphorylation of their targets, including KIF3A, KIF3B, and IFT74. **(B)** CILK1 and MAK are phosphorylated at Thr-157 and activated by KATNIP and CCRK, whereas they are dephosphorylated at Thr-157 and inactivated by PP5. In contrast, CILK1 and MAK are proposed to be phosphorylated at Tyr-15 and inactivated by FGFRs.

CILK1 phosphorylates Thr-674 in the C-terminal tail of KIF3A, a subunit of kinesin-2, at the ciliary tip (Chaya et al., 2014; Oh et al., 2019). MAK also phosphorylates KIF3A in retinal photoreceptor cells (Chaya et al., 2024), suggesting that CILK1 and MAK facilitate the disassembly of IFT complexes through the phosphorylation of KIF3A Thr-674 at the ciliary tip. In contrast, MEFs carrying a Thr-to-Ala mutation at residue 674 on KIF3A exhibit slightly elongated cilia without affecting the ciliary localization of IFT88, an IFT-B component (Gailey et al., 2020), showing that CILK1 and MAK may have other target(s) in addition to KIF3A. In *Chlamydomonas*, Ser-663 phosphorylation of the kinesin-2 motor subunit FLA8, an ortholog of KIF3B, is required for the IFT turnaround process at the flagellar tip (Liang et al., 2014). This residue is located within a consensus amino acid sequence for phosphorylation by CILK1 and MAK, which is evolutionarily conserved among species, implying that the IFT turnaround at the ciliary tip is mediated by phosphorylation of KIF3B in addition to KIF3A by CILK1 and MAK in vertebrates (Figure 2A). In *C*. *elegans*, DYF-5 reduces the binding affinity between tubulin and IFT-B components IFT74/81 by phosphorylating IFT74, proposing a model in which DYF-5-mediated phosphorylation of IFT74 promotes tubulin unloading from anterograde trains at the ciliary tip (Figure 2A) (Jiang et al., 2022). Further investigations are needed to clarify the downstream regulatory mechanisms of the IFT turnaround process executed by CILK1 and MAK.

## Physiological and pathophysiological roles of ciliary kinases CILK1 and MAK

*Cilk1*-deficient mice exhibit neonatal lethality accompanied with developmental abnormalities observed in multiple organs and tissues including the bone, lung, kidney, intestine, esophagus, brain, retina, and inner ear (Fu et al., 2019; Yang et al., 2021). In humans, homozygous loss-of-function mutations in the *CILK1* gene cause endocrine-cerebro-osteodysplasia (ECO) syndrome, an autosomal recessive ciliopathy characterized by neonatal lethality with multiple developmental defects involving the endocrine, cerebral, and skeletal systems (Lahiry et al., 2009; Oud et al., 2016), as well as short rib-polydactyly syndrome (SRPS), an autosomal recessive ciliopathy exhibiting perinatal lethality with short ribs, shortened and hypoplastic long bones, polydactyly, and multiorgan system abnormalities (Paige Taylor et al., 2016). In addition, heterozygous variants of the *CILK1* gene are linked to juvenile myoclonic epilepsy (Bailey et al., 2018). In contrast, *Mak*-deficient mice are viable and fertile without obvious developmental defects, but exhibit progressive retinal photoreceptor degeneration (Omori et al., 2010). Consistent with this, mutations in the human *MAK* gene lead to autosomal recessive retinitis pigmentosa (RP), a retinal degenerative disease characterized by photoreceptor degeneration (Ozgul et al., 2011; Tucker et al., 2011). Although the phenotypic differences between *Cilk1*-deficient and *Mak*-deficient mice suggest distinct roles of CILK1 and MAK *in vivo*, a recent study demonstrated genetic interactions between *Cilk1* and *Mak* in retinal photoreceptor cells (Chaya et al., 2024). It remains to be determined whether CILK1 and MAK play overlapping or distinct roles in other cell types, tissues, and organs.

## Regulatory mechanisms of ciliary kinases CILK1 and MAK activities

The phosphorylation of CILK1 and MAK at Thr-157 and Tyr-159 in the TDY motif is critical for their kinase activity (Fu et al., 2005; Fu et al., 2006; Wang and Kung, 2012). Cell cycle-related kinase (CCRK), also known as cyclin-dependent kinase 20 (CDK20), phosphorylates CILK1 and MAK at Thr-157 *in vitro* and in mouse retinal photoreceptor cells (Fu et al., 2006; Wang and Kung, 2012; Chaya et al., 2024). Inhibition of CILK1 Thr-157 phosphorylation leads to cilia elongation and accumulation of IFT88 at the ciliary tips in cultured cells (Yang et al., 2013; Nakamura et al., 2020). Similar to the loss of *Cilk1* or *Mak*, *Ccrk* deficiency results in cilia elongation and accumulation of IFT-A and IFT-B components at the ciliary tips in cultured cells (Snouffer et al., 2017; Noguchi et al., 2021). *Ccrk*-deficient mice exhibit multiple abnormalities associated with ciliopathies and dysregulation of Hedgehog signaling, including neural tube patterning defects, polydactyly, and malformation of the lungs and eyes (Snouffer et al., 2017; Lupu et al., 2018; Lee and Ko, 2020). Loss of *Ccrk* causes severe retinal degeneration, resembling that observed in *Cilk1* and *Mak-double-knockout* retinas (Chaya et al., 2024). Based on these observations, the CCRK-CILK1/MAK kinase signaling axis was proposed to play a crucial role in the regulation of the IFT turnaround process (Figure 2B). CCRK physically and functionally interacts with BROMI, also known as TBC1D32 (Ko et al., 2010). Mutations in the human *BROMI* gene cause ciliopathies (Adly et al., 2014), suggesting that CCRK-CILK1/MAK kinase signaling also occurs in humans. In contrast to CCRK, fibroblast growth factor (FGF) signaling negatively regulates CILK1 activity through FGF receptors (FGFRs)-mediated phosphorylation of CILK1 (Figure 2B) (Kunova Bosakova et al., 2019). FGF treatment of cultured cells modulates cilia length via CILK1. FGFR1, FGFR3, and FGFR4 interact with CILK1. FGFR3 phosphorylates CILK1 and MAK. CILK1 is phosphorylated by FGFR3 at Tyr-15, which is conserved in CILK1 and MAK. In addition, the basal body protein KATNIP (Sanders et al., 2015), also known as KIAA0556, and the protein phosphatase PP5 have been suggested to be modulators of CILK1 activity (Figure 2B). Overexpression of KATNIP increases protein levels and Thr-157 and Tyr-159 phosphorylation of CILK1 in cultured cells (Turner et al., 2023). PP5 dephosphorylates CILK1 at Thr-157 *in vitro* and in cultured cells (Fu et al., 2006). Although CCRK and KATNIP promote phosphorylation of CILK1 and MAK at Thr-157, the functional relationship between CCRK and KATNIP remains unclear. To what extent KATNIP- and PP5-mediated regulation of CILK1 and MAK contributes to cilia formation and function awaits future research.

## CILK1 and MAK as potential therapeutic targets

Recently, CILK1 and MAK have emerged as potential therapeutic targets for the treatment of ciliopathies and age-related obesity. Overexpression of MAK and CILK1 rescued ciliary defects observed in *Cilk1*-deficient cultured cells and *Mak*-deficient retinal photoreceptor cells, respectively (Chaya et al., 2024). Administration of a small-molecule inhibitor of FGFRs, which negatively regulates CILK1 activity, suppresses retinal degeneration observed in RP model *Mak*-deficient mice (Ozgul et al., 2011; Tucker et al., 2011; Kunova Bosakova et al., 2019; Chaya et al., 2024). Overexpression of CILK1, MAK, and CCRK, and treatment with an FGFR inhibitor rescued ciliary defects in cultured cells knocked down for *Dync2li1*, a ciliopathy gene encoding cytoplasmic dynein-2 light intermediate chain 1 (Taylor et al., 2015; Chaya et al., 2024). These observations suggest that promotion of disassembly of anterograde IFT trains at the ciliary tips through CILK1 and MAK activation can ameliorate ciliopathies manifesting defects in the turnaround process and retrograde transport.

The G protein-coupled receptor melanocortin-4 receptor (MC4R) localizes and functions at the neuronal primary cilia (Siljee et al., 2018; Wang et al., 2021). MC4R receives α-melanocyte stimulating hormone and agouti-related peptide in the hypothalamus, and plays essential roles in long-term regulation of energy homeostasis (Krashes et al., 2016). In humans, heterozygous loss-of-function mutations in *MC4R* are the most common monogenic cause of obesity (Vaisse et al., 1998; Vaisse et al., 2000; Lubrano-Berthelier et al., 2006). The length of MC4R-positive cilia in hypothalamic neurons decreases with age, which is promoted by overnutrition (Oya et al., 2024). Shortening of MC4R-positive cilia in hypothalamic neurons disrupts the regulation of energy homeostasis, resulting in obesity (Oya et al., 2024). Knockdown of *Cilk1* in hypothalamic neurons increases MC4R-positive cilia length and reduces body weight gain in rats fed a high-fat diet (Oya et al., 2024), suggesting inhibition of CCRK-CILK1/MAK kinase signaling as a therapeutic strategy for age-related obesity. Given that loss-of-function of *Cilk1* inhibits the IFT turnaround process at ciliary tips, how *Cilk1* knockdown in hypothalamic neurons can improve ciliary function to suppress obesity awaits future studies.

## Conclusion

It has become clear that IFT is regulated by several serine-threonine kinases. In particular, the identification and functional characterization of the ciliary kinases CILK1 and MAK have unraveled the molecular mechanisms underlying the IFT turnaround process and their physiological and pathophysiological significance. Recently, CILK1 and MAK have emerged as potential therapeutic targets for human diseases including ciliopathies and age-related obesity. Genetic and pharmacological activation of CCRK-CILK1/MAK kinase signaling can suppress ciliary abnormalities caused by the knockdown of a gene encoding a cytoplasmic dynein-2 component. Patients with mutations in the genes encoding IFT-A, cytoplasmic dynein-2 components, and CILK1 exhibited a similar spectrum of ciliopathy symptoms (Mitchison and Valente, 2017), suggesting a functional relationship among IFT-A, cytoplasmic dynein-2, and CILK1. Understanding how CILK1 and MAK regulate the IFT turnaround process by phosphorylating the downstream target(s) could reveal the extent to which the activation of CCRK-CILK1/MAK kinase signaling can be more generally applicable to treat human ciliopathies.
